# Common Characteristics Between Frailty and Myotonic Dystrophy Type 1: A Narrative Review

**DOI:** 10.14336/AD.2024.0950

**Published:** 2025-08-29

**Authors:** Joana Garmendia, Garazi Labayru, Philipe de Souto Barreto, Itziar Vergara, Adolfo López de Munain, Andone Sistiaga

**Affiliations:** ^1^Department of Clinical and Health Psychology and Research Methodology, Psychology Faculty, University of the Basque Country (UPV/EHU), Donostia-San Sebastián, Gipuzkoa, Spain.; ^2^Centro de Investigación Biomédica en Red sobre Enfermedades Neurodegenerativas (CIBERNED), Institute Carlos III, Madrid, Spain.; ^3^Neuroscience Area, Biogipuzkoa Health Research Institute, Donostia-San Sebastián, Gipuzkoa, Spain.; ^4^Institute on Aging, Toulouse University Hospital (CHU Toulouse), Toulouse, France.; ^5^Institut Hospitalo-Universitaire (IHU) HealthAge, Toulouse, France.; ^6^CERPOP UMR 1295, Inserm, Université Paul Sabatier, Toulouse, France.; ^7^Osakidetza Health Care Directorate, PC-IHO Research Unit of Gipuzkoa, Donostia-San Sebastián, Gipuzkoa, Spain.; ^8^Primary Care Group, Biogipuzkoa Health Research Institute, Donostia-San Sebastián, Gipuzkoa, Spain.; ^9^Red de Investigación en Cronicidad, Atención Primaria y Promoción de la Salud (RICAPPS), Spain.; ^10^Neurology Department, Donostia University Hospital, Donostia-San Sebastián, Gipuzkoa, Spain.

**Keywords:** Steinert’s disease;, Fried’s frailty phenotype;, accelerated aging, disease-related frailty, senescence, psychosocial factors

## Abstract

Myotonic dystrophy type 1 (DM1) is an inherited neuromuscular disorder often considered a model of accelerated aging due to the early appearance of certain age-related clinical manifestations and cellular and molecular aging markers. Frailty, a state of vulnerability related to aging, has been recently studied in neurological conditions but has received considerably less attention in neuromuscular disorders. This narrative review aims to describe 1) the common characteristics between Fried’s frailty phenotype criteria (muscular weakness, slow gait speed, weight loss, exhaustion/fatigue, and low physical activity) and DM1, and 2) the psychological and social factors potentially contributing to frailty in DM1. This review gathered evidence suggesting that DM1 patients meet four of the five frailty phenotype criteria. Additionally, longitudinal studies report the deterioration of these criteria over time in DM1. Patients also exhibit psychological/cognitive and social factors that might contribute to frailty. Monitoring frailty criteria in the DM1 population could help to implement timely preventions and interventions to reduce the disease burden and severity of frailty symptoms.

## 1. Introduction

Myotonic dystrophy type 1 (DM1), also known as Steinert’s disease, is considered a rare condition; however, it is the most common form of muscular dystrophy in adults. Clinical-based prevalence studies estimate that DM1 affects 5 to 20 per 100,000 individuals globally [1]. It is a genetic disorder inherited in an autosomal dominant manner and characterized by molecular instability, which correlates with earlier disease onset and greater severity [2, 3]. DM1 is classically characterized by progressive muscular weakness, atrophy, and myotonia, alongside other systemic manifestations affecting various body systems, including the central nervous system (CNS), cardiac, endocrinal, ophthalmological, and gastrointestinal systems [1, 4]. DM1 patients can be classified into different phenotypes based on the age of disease onset [5]. Generally, the earlier the signs and symptoms appear, the more severe the disease. Some phenotypes include intellectual disability, leading to greater psychosocial challenges [6-8].

DM1 has been proposed as a model of premature or accelerated aging, which, as defined by Margolick and Ferrucci (2015) [9], is characterized by anatomical and functional manifestations and underlying mechanisms that mirror those observed in normal aging but occur prematurely or earlier than typically expected. In the DM1 population, patients prematurely present age-related clinical manifestations (e.g., cataracts, frontal baldness, muscle weakness, cognitive impairment, metabolic dysfunction). Cellular findings in DM1 also resemble the hallmarks of aging, including senescence inducers (telomere shortening, mitochondrial dysfunction, and oxidative stress) and senescence biomarkers (cell cycle inhibitors, senescence-associated secretory phenotype, chromatin reorganization, and microRNA), along with molecular features similar to those of segmental progeroid disorders [10-14].

In an aging population, frailty emerges as a crucial concept, defined as a state of vulnerability between robustness and disability associated with age. It is considered a potentially reversible condition [15] resulting from the cumulative decline of several physiological systems and vulnerability to internal and external stressors [16]. The frailty phenotype was first characterized by Fried [17] and includes at least three criteria: muscle weakness, slow walking speed, unintentional weight loss, exhaustion or fatigue, and low physical activity. In recent years, researchers have acknowledged the contribution of other psychological and social factors to frailty, leading to a more comprehensive and multidimensional approach to understanding the construct [18-21].

Traditionally studied in geriatrics, the concept of frailty has extended into other medical disciplines as a measure of an individual’s risk profile [22]. Fried and colleagues [17] proposed two distinct paths leading to frailty: age-related and disease-related frailty, with the latter arising from chronic or acute medical events. Angioni and colleagues [23] supported this hypothesis, suggesting clinical differences and trajectories between the two paths. Neurological conditions, such as Alzheimer’s disease, Parkinson’s disease, and multiple sclerosis, have emerged as significant contributors to disease-related frailty, with a high prevalence of frailty observed in these conditions [24, 25]. Frailty itself serves as a risk factor for disease progression and increased vulnerability to adverse medical events [26]. Additionally, frailty not only increases the risk of developing neurological disorders [27] but also influences their clinical presentation and phenotypical expression, being associated with adverse health outcomes [24, 28] alongside certain disease-specific features [29, 30]. However, there is very limited literature available on frailty within neuromuscular disorders [31, 32].

Due to the potential accelerated aging process in DM1 and the possible increased risk of frailty, this narrative review aims to analyze the common characteristics between Fried’s frailty phenotype criteria and DM1. Additionally, considering the CNS alteration and psychosocial factors affecting DM1 patients, the second aim of this review is to address the psychological and social factors potentially contributing to frailty in DM1.

## 2. Method

Articles were searched in MEDLINE (PubMed), Scopus, and Web of Science databases until August 2024. To align the review with the stated objectives, frailty was defined using Fried’s frailty phenotype criteria. To ensure the inclusion of the most recent studies since the establishment of Fried’s criteria in 2001, this review considered works published from 2001 to the present (August 2024). The search strategy included the terms “DM1” or “myotonic dystrophy,” combined with keywords related to Fried’s frailty phenotype criteria (muscle weakness, gait speed, weight loss, exhaustion/fatigue, and physical activity) for the first aim, and psychological and social contributors to frailty for the second aim. To further expand the range of studies, a forward and backward snowballing approach was employed [33].

Inclusion criteria for this narrative review were limited to papers published in English or Spanish. Only human studies involving adult populations, both cross-sectional and longitudinal, were included. Interventional studies were considered only in exceptional cases. Exclusion criteria included case reports, conference papers, studies on myotonic dystrophy type 2, and any research studies beyond the scope of this review.

The included articles were screened by reading the abstracts, and relevant articles were comprehensively reviewed.

## 3. Results and discussion

This narrative review is divided into two sections: the first section focuses on the frailty phenotype criteria, while the second section addresses the psychological and social factors potentially contributing to frailty. As of the date of this review, there are no studies on frailty in the DM1 population. In this review, each component or factor of frailty is briefly described within the context of aging and geriatrics. Subsequently, a comprehensive examination is conducted within the DM1 population, discussing the available literature.

### 3.1. Frailty phenotype and DM1

The studies addressing the common characteristics between Fried’s frailty phenotype criteria and DM1 are summarized in Table 1 and are discussed below. Overall, the findings and conclusions from the reviewed articles were consistent and aligned, showing no discrepancies, which strengthens the presented evidence.

**Table 1:** Summary of the common characteristics between frailty phenotype criteria and DM1.

Fried’s frailty phenotype criteria	DM1	Transversal studies in DM1	Studies	Longitudinal studies in DM1	Studies
**Muscle weakness:** grip strength in the lowest 20% at baseline, adjusted for gender and body mass index.	✔	-Myotonia -Low grip strength	(Hogrel et al., 2017; LoRusso et al., 2018; Moxley et al., 2007; Ozimski et al., 2021; Sugimoto et al., 2022; Thornton, 2014)	-Progressive muscle atrophy and weakness -Loss of muscle strength	(Gagnon et al., 2018; Hammarén et al., 2015; Raymond et al., 2017; Roussel et al., 2021; Sedehizadeh et al., 2017)
**Slow gait speed:** The slowest 20% of the population, based on time to walk 15 feet, adjusting for gender and standing height.	✔	-Gait disturbances: altered gait speed, step length and frequency, and cadence	(Bachasson et al., 2016; Galli et al., 2012; Kim et al., 2020; Pucillo et al., 2018; Wiles, 2005)	-Deterioration of gait parameters -Increase in walking difficulties	(Hammarén et al., 2015)
**Weight loss:** weight loss, unintentional, of ≥10 pounds in prior year	-	-No specific studies -Two phenotypes: atrophic and overweight (obesity) -Increased fat-mass (visceral adiposity) -Decreased fat-free mass -Possible contributor: dysphagia (prevalence 55%)	(Gutiérrez et al., 2020; Joosten et al., 2023; Kikuchi et al., 2022; Peric, Bozovic, et al., 2019; Pruna et al., 2011; Sedehizadeh et al., 2017)	-No specific studies -Increase on total fat-mass and a decrease in total fat-free mass -Decrease in both trunk fat-mass and fat-free mass (possible weight loss)	(Kikuchi et al., 2022; Sedehizadeh et al., 2017)
**Exhaustion/fatigue:** as indicated by self-report of exhaustion.	✔	-High prevalence of fatigue (50-90%)	(Erokhina et al., 2024; Heatwole et al., 2012; Kalkman, 2005; Laberge et al., 2020; Landfeldt et al., 2019; Menting et al., 2018; Peric, Bjelica, et al., 2019),	-Increase in fatigue levels and/or proportion of patients with fatigue over time	(Kalkman, 2005; Laberge et al., 2020; Peric, Bjelica, et al., 2019)
**Low physical activity:** A weighted score of kilocalories expended per week, based on each participant's report.	✔	-Less physically active than healthy controls -Need assistance or have difficulties performing physical activity	(Gagnon et al., 2007, 2013; Garmendia et al., 2023; Kierkegaard et al., 2011; Knak et al., 2020; Landfeldt et al., 2020; Van Heugten et al., 2018; Wiles, 2005)	-Decrease in physical activity over time	(Raymond et al., 2019)

*Note.* DM1 = Myotonic dystrophy type 1

#### 3.1.1. Muscle weakness

Muscle weakness, a key characteristic of the frailty phenotype, is operationalized as low grip strength. Research suggests that it peaks in early adulthood, remains stable through midlife, and subsequently declines with age, typically starting at the age of 50 [34]. In older adults, grip strength is a predictor of overall strength and function [35] and is a significant indicator of current health status and physical, functional, and psychological performance [35-38]. Additionally, it is considered a mediator in the relationship between muscle mass and frailty [39] and a predictor of morbidity and mortality [40, 41]. Although grip strength is a key component of muscle weakness, other aspects, such as lower limb strength, are also worth considering in the context of frailty.

DM1, a progressive neuromuscular condition, is typically characterized by progressive muscle weakness, atrophy, and handgrip myotonia, which refers to impaired muscle relaxation after voluntary contraction [42], leading to weak grip strength. The muscular involvement in DM1 follows a symmetric and distal pattern, gradually progressing to proximal muscles. It commonly causes distal weakness in the upper (forearms, hands) and lower (finger flexors, ankle plantar flexors, ankle dorsiflexors) limbs, with the face, jaw, and neck muscles being the most severely affected muscle groups [4, 43].

Myotonia and distal muscular weakness are among the first signs of DM1 [1] and are frequently observed symptoms [44], typically evident during maximal grip contractions. Regarding the natural course of myotonia in DM1, certain studies suggest that the myotonia sign tends to decrease with age [11], likely due to the aggravation of muscular weakness, resulting in insufficient strength to manifest contraction and relaxation signs.

Progressive skeletal muscle atrophy and weakness can reduce muscle strength and endurance [42]. In line with this observation, DM1 patients exhibit weak grip strength and different grip force relaxation compared to healthy controls (HC) [45, 46]. Myotonia and grip strength are associated with the molecular defect of the disease (CTG expansion size) [44-47] and serve as markers of disease severity. Additionally, muscular weakness is linked to an increased risk of falls and fractures in DM1 [48, 49], with the incidence of these events being high and age-related in this population [50].

Longitudinal studies have shown a significant loss of muscle strength in various muscle groups [51-53] and a significant decline in grip strength [54, 55].

#### 3.1.2. Slow gait speed

Another characteristic of the frailty phenotype is slow gait speed, which is a significant predictor of the risk of injurious falls, which can potentially lead to medical complications, such as fractures. Other gait parameters are also related to adverse clinical, cognitive, and physical health outcomes [56].

The progressive deterioration of muscles in DM1 patients can profoundly affect gait and balance, leading to commonly observed disturbances in these areas. Various studies in DM1 have reported altered gait parameters, including altered gait speed, step length and frequency, and cadence [57-60]. These gait deficiencies are closely associated with an increased risk of falls and fractures in DM1 patients (Bachasson et al., 2016; Galli et al., 2012; Kim et al., 2020; Pucillo et al., 2018), with the prevalence of falls ranging from 30-70% and fall-related fractures from 11-17% [49, 60, 63]. Additionally, individuals with DM1 experience a twofold risk of falling compared to healthy controls aged over 65.

Furthermore, a 5-year follow-up study noted significant deterioration of gait parameters and muscle strength, balance, and performance-based measures, resulting in a significant increase in reported walking difficulties [52].

#### 3.1.3. Weight loss

Unintentional weight loss or shrinking is another common feature contributing to the frailty phenotype. In the context of aging, weight loss often occurs due to reduced food intake [64]. This weight loss can be attributed to various age-related changes, such as loss of appetite, gastrointestinal alterations, metabolic changes, altered taste and smell, and psychological and social factors [65]. Additionally, illness and the effects of medications can further contribute to weight loss. This weight loss can lead to nutritional deficiencies and is often accompanied by sarcopenia, defined as the loss of skeletal muscle mass and function due to aging [66].

In the case of DM1, two distinct phenotypes can be found in patients: the atrophic phenotype and the overweight or obesity phenotype, with the latter being the most predominant. Indeed, some studies have shown that overweight and obesity are prevalent in DM1, affecting 50% and 25-50% of patients, respectively [67, 68]. Furthermore, studies on body composition in DM1 have reported alterations such as increased fat mass (FM) and visceral obesity and decreased fat-free mass (FFM), even in patients with a normal body mass index [55, 67, 69, 70]. These changes could be due to physical inactivity, poor dietary habits, and metabolic alterations such as insulin resistance [71]. These findings suggest the potential presence of both sarcopenic obesity (co-existence of sarcopenia and obesity) and osteosarcopenia (co-existence of sarcopenia and osteoporosis) in DM1 patients [72, 73].

On the other hand, concerning the atrophic phenotype, there is a lack of literature specifically addressing weight loss in DM1 patients. However, certain clinical features of the condition may contribute to unintentional weight loss. One significant feature is the high prevalence of gastrointestinal dysfunction in DM1 patients [74, 75], which includes symptoms such as dysphagia (difficulty in swallowing), abdominal pain, constipation, and diarrhea. Dysphagia, present in approximately 55% of DM1 patients [76], is not only a significant risk factor for aspiration pneumonia but can also lead to malnutrition and unintentional weight loss [77], further exacerbating nutritional deficiencies and muscle wasting associated with physical frailty.

While no longitudinal studies have addressed weight loss specifically in DM1, certain studies on body composition have yielded inconsistent results. One study reported a significant increase in total FM and a decrease in total FFM [55]. In contrast, another study with a similar follow-up period showed a significant reduction in both trunk FM and FFM [69], suggesting the possibility of weight loss.

#### 3.1.4. Exhaustion/fatigue

Exhaustion or fatigue is considered a central component of frailty and is characterized by poor endurance and energy. The Diagnostic and Statistical Manual of Mental Disorders, 5th Edition, [78] defines fatigue as a state associated with reduced physical and/or mental resources, ranging from general lethargy to a specific burning sensation in the muscles induced by work. Although measures of fatigue in frailty vary, most refer to fatigue as a general feeling of tiredness or diminished energy and endurance rather than muscle fatigability [79, 80].

Fatigue is highly prevalent in DM1, affecting 50-90% of individuals with the condition [81-86]. This prevalence is higher than in other neuromuscular disorders [87]. Fatigue in DM1 is not necessarily related to muscular impairment, as patients with mild muscle symptoms can experience severe fatigue.

In DM1 patients, fatigue has been found to correlate with disease severity markers, such as muscular impairment and molecular defect (CTG), as well as psychological features and quality of life [82, 85, 86, 88]. Moreover, fatigue is commonly associated with another CNS-related symptom, daytime excessive sleepiness [82], a common feature within this population. Notably, in older adults, daytime sleepiness has been associated with sarcopenia, an increased risk of falls, age-related comorbidities, and mortality [89, 90].

Although inconclusive, longitudinal studies suggest that fatigue levels and the proportion of patients with fatigue tend to increase over time and are also associated with age [82, 85, 87].

#### 3.1.5. Low physical activity

Frailty phenotype is also characterized by low levels of physical activity, which is considered one of the key determinants of frailty [91]. Physical activity tends to decrease with aging [92], and physical inactivity and sedentary behavior are prevalent among older adults [93]. Lack of physical activity is considered a risk factor for incident frailty [91, 94, 95] and is linked to reduced muscle mass and strength [96], along with increased risk of disease, disability, and mortality [97].

Studies have reported that DM1 patients encounter significant physical activity or fitness challenges, often requiring assistance [98-102]. Research indicates that DM1 patients are less physically active compared to healthy adults or the reference population, as measured by objective and subjective techniques [60, 103], with half of the studied patients meeting the minimum requirements for WHO-recommended physical activity [104]. Sedentary DM1 patients tend to present worse functional performance, suggesting that physical activity could have a protective effect on the loss of functional ability [53].

In the study by Knak and colleagues [104], the low physical activity levels observed in DM1 patients are attributed to several factors, including the high prevalence of muscle weakness, fatigue, apathy, and cognitive impairment, along with other challenges such as financial difficulties or limited access to facilities. However, their study identified education as the only significant predictor of physical activity among DM1 patients [104].

In this regard, a longitudinal study reported a significant decrease in physical activity over a nine-year period among DM1 patients, with levels falling below those of healthy adults aged 55-64 at baseline [105]. The authors suggested that these changes reflect not only aging effects but also disease-specific factors inherent to DM1.

An interventional study involving cognitive behavioral therapy combined with optional graded exercise in DM1 patients significantly improved physical activity levels and exercise capacity and lowered fatigue compared to standard care alone [106]. Another study found that an activity-stimulating behavioral intervention improved the capacity for activity and participation in the intervention group, while the standard care group saw a decline. However, physical activity levels remained stable in both groups [107].

Regular physical activity is essential for maintaining muscle strength [108]. Therefore, low physical activity in DM1 could contribute to muscle disuse, resulting in decreased strength and potentially leading to further muscle atrophy. This situation exacerbates the risk of poor health outcomes, disability, and frailty.

### 3.2. Psychosocial factors contributing to frailty in DM1

#### 3.2.1. Psychological factors

While the concept of psychological frailty lacks a universally accepted definition, a recent scoping review [109] identified common psychological components contributing to frailty. These components include mood alterations (depression, sadness), cognitive alteration (cognitive deficits, dementia), other mental health concerns (anxiety, poor coping, and loneliness), and fatigue-related problems (including fatigue, exhaustion, and loss of energy).

Various studies have reported a bidirectional relationship between psychological/cognitive function and physical frailty [110-113]. Both psychological symptoms and cognitive impairment or reduced cognitive reserve can potentially increase vulnerability to stressors and the risk of adverse health outcomes, such as neurodegenerative processes [114-116].

In DM1, alongside muscular impairment, patients often present central nervous system involvement, including brain alterations, cognitive impairment, and symptoms such as apathy, fatigue, and daytime sleepiness [117]. Concerning cognition, these patients tend to have a slightly diminished IQ and cognitive deficits across various domains [118, 119], including social cognition [120], and longitudinal studies have indicated a slow progressive decline in cognitive function [121-128], suggesting a neurodegenerative process. However, it remains challenging to determine whether these cognitive deficits in DM1 are primarily associated with disease-related alterations in early-life stages (e.g., neurodevelopmental) or are linked to an additional neurodegenerative process derived from aging.

Regarding the psychopathological profile of DM1 patients, these individuals often face chronic and progressive conditions that evoke challenging emotions such as frustration, anxiety, and sadness [8]. Various studies, including a meta-analysis [129-131], have revealed mild psychopathological problems in DM1 patients, with higher rates of mood disorders (19% depression, 17% anxiety, and 55% apathy), somatic disorders, and personality disorders compared to the healthy population. Apathy emerges as the most prevalent psychological symptom in DM1, and interestingly, recent studies in the general older adult population have linked apathy to incident frailty and disability, with apathetic individuals presenting a higher risk of being classified as pre-frail or frail [132-134].

#### 3.2.2. Social factors

Social factors are also significant contributors to frailty. Frailty is associated with social vulnerability, characterized by a lack of essential social resources needed to fulfill basic social requirements. This vulnerability includes increased loneliness and social isolation, inadequate social support, reduced social participation, and declining self-management abilities [135, 136]. Social factors constitute a crucial component associated with an elevated risk of adverse medical events and mortality.

Studies consistently highlight challenges in daily activities and disrupted social participation in DM1 patients, including difficulties in recreational activities, social interactions, and employment [98-102]. Follow-up studies indicate a progressive decline in both daily and social activities in DM1 patients [105].

Other studies in DM1 also identified unmet needs in social and personal care despite adequate medical attention [137], along with indicators of socioeconomic deprivation, such as poor academic achievement, high unemployment rates, low family income, and a greater need for social assistance compared to the healthy population [138]. Determinants of this social dysfunction in DM1 include disease-related factors (disease duration, impaired social cognition, apathy, fatigue, and muscular impairment) and environmental factors (limited social network, stigma, low educational attainment, and unemployment), all contributing to difficulties in the social domain [99, 105, 139-141].

## 4. Conclusions and future directions

This narrative review is the first study to explore the common clinical characteristics between frailty and DM1, focusing on the frailty phenotype criteria as characterized by Fried [17], along with the psychosocial factors potentially contributing to frailty.

The main finding of this review is the substantial overlap of the Fried’s frailty phenotype criteria with the symptomatology characteristic of DM1. Evidence has been gathered to suggest that DM1 patients meet at least four frailty phenotype criteria. Specifically, the literature confirms that DM1 patients commonly experience muscular weakness [45, 46], slow gait speed [57-60], fatigue/exhaustion [81-85], and reduced physical activity [60, 103, 104]. However, there is no conclusive evidence supporting unintentional weight loss in DM1. Although there is a tendency towards overweight and obesity in these patients [67, 68], this does not rule out the possibility of the coexistence of overweight/obesity and weight loss. Notably, DM1 patients present both low muscle mass and high adiposity [55, 67, 69], resembling characteristics observed in sarcopenic obesity [72].

In addition, longitudinal studies in DM1 have reported a deterioration of the frailty phenotype criteria over time. Specifically, muscle weakness tends to worsen progressively [42, 51, 53, 142], and gait parameters, including speed, significantly decline [52]. Additionally, fatigue levels have been observed to increase with age [82, 85, 87], and physical activity decreases significantly over time [105] in DM1.

Beyond the physical domain, psychosocial factors, including psychological, cognitive, and social factors, could also influence the risk profile of the DM1 population. DM1 patients often show slight cognitive deficits [118] and experience a gradual decline in cognitive function over time [121-125]. Psychopathological symptoms, particularly apathy, are prevalent among these patients [129, 130]. Additionally, DM1 patients encounter challenges in social functioning, including difficulties in daily activities, reduced social participation, and lower levels of education and employment [98-102], which tend to worsen progressively over time [105].

The primary challenge in studying frailty in DM1 lies in the potential overlap between frailty and DM1 symptomatology. Given the complex, multisystemic nature of the disease, it is difficult to conclusively attribute frailty to specific disease characteristics, the potential accelerated aging processes, or an interaction between both. As highlighted in this review, there is a notable absence of studies specifically addressing frailty in DM1. Consequently, one of the main limitations is that frailty could only be examined by separately analyzing the components of the frailty phenotype in DM1. Additionally, there is a potential bias since only articles meeting the inclusion criteria and deemed relevant to the objectives of this narrative review were included, which may have resulted in the omission of certain pertinent studies. It is also important to note that the reviewed studies were conducted on adult DM1 patients with varying phenotypes (based on age of onset), which should be considered when generalizing the conclusions. Finally, beyond the limitations of this review, it is crucial to recognize that the reviewed studies might have their own biases and limitations, such as small sample sizes, which are inherent constraints in the study of a rare disease like DM1.

Frailty is characterized by its potential reversibility in healthy older adults [15], highlighting the importance of its identification and management. However, it remains to be explored whether frailty symptoms could be reversed in clinical populations (or in the case of disease-related frailty), particularly in DM1 patients.

Studying frailty in DM1 could provide valuable insights for identifying vulnerable individuals at risk of adverse health outcomes and mortality. Moreover, monitoring and managing frailty in this clinical population could have important implications for the clinical care of these patients. Beyond the usual clinical approach of DM1 patients, identifying and stratifying them based on their frailty status could facilitate the implementation of targeted prevention and intervention programs for the most vulnerable individuals. These initiatives could aid in preventing, decelerating, and even reversing frailty, thereby potentially delaying the onset of disability in this population. Such efforts could be key in improving patients' health-related quality of life since a recent study reported that disease-related frailty might progress to disability more rapidly than age-related frailty [23].

Understanding how frailty impacts prognosis and mortality in DM1 is essential. Therefore, future research should focus on determining the prevalence of frailty in the DM1 population and its relationship with disease-specific features (molecular defect, muscular impairment, and multisystemic involvement), and adverse health outcomes. Studies addressing common biological mechanisms between DM1, and frailty would also be of particular interest. Additionally, longitudinal studies are needed to track the natural progression of DM1 and frailty components over time and address the effects of interventions on frailty. Such research would provide valuable insights into the impact of frailty on clinical manifestations.

In summary, this narrative review has identified physical, psychological, and social features in the DM1 population that align with or are associated with frailty and support the idea of an ongoing accelerated aging process. These findings suggest a potentially higher prevalence of frailty in DM1, which may contribute to an increased risk of adverse health outcomes and mortality. Insights gained from studying frailty in DM1 patients could inform the development of effective interventions to mitigate disease progression and prevent adverse outcomes and disability in vulnerable individuals.
